# Free water improves sodium mobilization in furosemide treated pigs after a hyperosmotic sodium load

**DOI:** 10.1186/s40635-025-00800-5

**Published:** 2025-08-21

**Authors:** Annelie Barrueta Tenhunen, Anders Larsson, Olav Rooyackers, Miklos Lipcsey, Michael Marks-Hultström

**Affiliations:** 1 Anaesthesiology and Intensive Care Medicine, Department of Surgical Sciences, Uppsala University, Uppsala, Sweden; 2 Clinical Chemistry, Department of Medical Sciences, Uppsala University, Uppsala, Sweden; 3 Division of Anesthesiology and Intensive Care, Department of Clinical Science, Intervention and Technology, Karolinska Institute, Stockholm, Sweden; 4 Integrative Physiology, Department of Medical Cell Biology, Uppsala University, Uppsala, Sweden

**Keywords:** Fluid management, Hypernatremia, Kidney function, Sodium excretion

## Abstract

**Background:**

Hypernatremia, a common electrolyte disorder in critically ill patients, induces a hyperosmotic state linked to increased mortality and metabolic stress. While loop diuretics such as furosemide are used for fluid management, their main effect is water excretion, often worsening hypernatremia. This study aimed to determine whether free water infusion enhances sodium excretion when combined with furosemide after a sodium chloride bolus. We also hypothesized that hyperosmolar hypernatremia stimulates protein degradation and urea synthesis.

**Results:**

Fourteen pigs (seven per group) received a sodium chloride bolus to induce hypernatremia (plasma Na⁺ > 150 mmol/L). One group received furosemide alone, while the other received furosemide plus free water to maintain normo-osmolality. Renal and metabolic parameters were analyzed over five hours.

Free water infusion significantly lowered plasma sodium levels (134 ± 4 vs. 150 ± 4 mmol/L, p = 1.2e−14) and increased total sodium excretion (99 ± 20 vs. 70 ± 18 mmol, p = 0.00056) and urine output (1860 ± 220 vs. 1200 ± 160 mL, p = 2.47e-05). Fractional sodium excretion was higher with free water (5.3 ± 1.1% vs. 3.5 ± 2.2%, p = 0.012). Plasma glutamine was elevated in the no-water group (1305 ± 209 vs. 1084 ± 110 µmol/L, p = 0.029), indicating greater metabolic stress.

**Conclusions:**

These results suggest that free water infusion enhances sodium clearance and reduces hypernatremia-induced metabolic alterations, supporting its potential role in fluid management strategies.

**Graphical Abstract:**

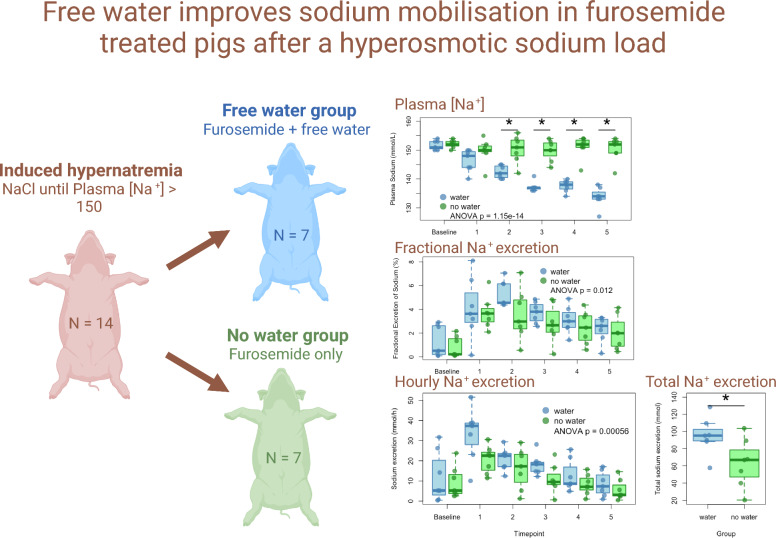

## Introduction

Hypernatremia, a common electrolyte disorder, is defined as a serum sodium concentration of more than 145 mmol/l. The condition represents a relative excess of sodium in relation to body water [1], either caused by a deficit of water or, by a gain of sodium. Since serum sodium with corresponding anions constitutes the main determinant of plasma osmotic activity, hypernatremia always mirrors a hyperosmolar, hypertonic state [2, 3].

Serum osmolality and water homeostasis are, under normal conditions, maintained within a tight range via regulation of thirst and secretion of arginine vasopressin [4]. Fluid resuscitation and maintenance fluids contain considerable amounts of sodium, this, in combination with impaired thirst and/or water access renders the critically ill patient prone to develop hypernatremia, often implying both volume and sodium overload [5–9]. In critical illness, hypernatremia is associated with a poor outcome, including increased length of stay and mortality [5, 10, 11].

Hypernatremia exerts a hyperosmotic, hypertonic stress on cells, leading to the activation of cellular adaptive mechanisms to counteract the loss of cell volume, such as ion transport and production of organic osmolytes [4]. The hypertonic condition leads to a catabolic state with protein degradation [12–14], potentially compounding the hyperosmotic state with additional organic osmolytes in the form of urea and glucose. As we have previously shown, hyperosmolality with increased urea levels is associated with mortality [12, 15].

The excretion of sodium is the net result of glomerular filtration rate and total tubular reabsorption, a balance that is primarily mediated via arterial pressure, sympathetic tone, the renin–angiotensin–aldosterone system and atrial natriuretic peptides [16]. Furosemide inhibits reabsorption and, by disrupting the counter-current mechanism, leads to a predominant increase in water excretion over electrolytes. However, it also results in a net increase in the excretion of sodium, chloride, potassium, magnesium, and calcium [17]. The infusion of hypertonic saline stimulates thirst [18], but whether the extra water is used by the kidney to excrete sodium and how the kidney handles sodium overload in relation to water is still under debate [19]. While loop-diuretics are often used for the treatment of fluid overload in the intensive care setting, their effect on outcome, such as mortality and serious adverse events, is uncertain [20]. Currently, furosemide is investigated for fluid removal in the intensive care setting [21].

We hypothesized that maintaining normo-osmolality using free-water infusion would enhance sodium excretion when used in conjunction with administration of furosemide in pigs after a sodium chloride bolus. To administer free water in addition to loop-diuretics might prevent dehydration induced ADH secretion and support ongoing natriuresis without concomitant activation of the renin–angiotensin–aldosterone system. Further, we hypothesized that hyperosmolar hypernatremia, as a sole insult, would stimulate protein degradation and, in turn, urea synthesis.

## Materials and methods

### Animals and ethics statements

The study was approved by the Animal Ethics Committee in Uppsala, Sweden (5.8.18-08592-2019/DOUU-2022-010) and was performed at the Hedenstierna Laboratory, Uppsala University, Sweden. The animals were handled in strict accordance with the National Institute of Health guide for the care and use of Laboratory animals [22] and reported according to the ARRIVE 2.0 guidelines. After premedication, the animals received continuous intravenous anesthesia and analgesia throughout the experiment. All measures were taken to minimize distress and suffering in the animals.

### Anesthesia and instrumentation

Fourteen pigs of both sex (Sus scrofa domesticus) of mixed Swedish Hampshire and Yorkshire breeds (mean weight 30.0 ± 1.6 kg) were premedicated with Zoletil Forte® (tiletamine and zolazepam) 6 mg/kg and Rompun® (xylazine) 2.2 mg/kg i.m. After adequate sedation was obtained, the animals were placed in supine position and received an i.v. bolus of fentanyl of 5–10 µg/kg through an intravenous catheter in an auricular vein. Thereafter, anesthesia was maintained with ketamine 30 mg/kg/h, midazolam 0.1–0.2 mg/kg/h and fentanyl 4 µg/kg/h, as a continuous infusion during the entire experiment. After the adequate depth of anesthesia was assured by the absence of reaction to pain stimulus between the front hooves the muscle relaxant rocuronium 2.5 mg/kg/h was added, in order to avoid spontaneous breathing.

The animals were under deep anesthesia during the whole experiment (five hours of furosemide-infusion), including euthanasia (100 mmol KCl i.v.). If signs of distress or reaction to pain stimulus were noted, bolus doses of 100 µg fentanyl were administered i.v.

The animals were tracheostomized and a tube of an internal diameter of eight mm (Mallinckrodt Medical, Athlone, Ireland) was inserted in the trachea. Thereafter, volume-controlled ventilation (Servo I, Maquet, Solna, Sweden) was maintained with settings as follows: respiratory rate (RR) 25/min, tidal volume (V_T_) 8 ml/kg, positive end-expiratory pressure (PEEP) 5 cmH_2_O, and inspired oxygen concentration (F_I_O_2_) 0.3.

A triple-lumen central venous catheter and a pulmonary artery catheter were inserted via the right jugular vein. An arterial catheter was inserted for blood pressure measurement and blood sampling via the right carotid artery. Via the right femoral artery, a PiCCO (Pulse index continuous cardiac output) catheter (Pulsion, Munich, Germany) was inserted for the estimation of stroke volume variation (SVV). Blood gas analyses were performed immediately after sampling on an ABL 800 analyzer (Radiometer, Copenhagen, Denmark). A midline mini-laparotomy was carried out for catheterization of the bladder for urinary drainage.

### Study protocol

Directly after the establishment of a peripheral venous catheter, a venous blood sample was drawn for blood gas analysis and determination of baseline sodium level in plasma. At this timepoint, an iohexol infusion (0.5 ml/h with a concentration of 350 mg/ml) was started after an initial bolus (0.5 ml) for measurement of glomerular filtration rate (GFR). Hypertonic saline (5%) was administered in repeated intravenous boluses until plasma levels of sodium were above 150 mmol/l. An equilibration period of ten minutes was respected before samples for repeated analyses were taken. After instrumentation and stabilization, baseline measurements and blood samples were collected and repeated hourly during the five hours of the experiment (timepoint 1–5). An infusion of furosemide (5 mg/h) was started with an initial bolus of 5 mg after baseline measurements, and the animals were assigned to two different groups; water and no water. The water group received free water to normalize plasma osmolality and compensate for urine production. This was given as 2 × the hourly diuresis of intravenous sterile water administration during the following hour, and adjusted for following hours throughout the protocol. The no water group received furosemide without additional fluids. If the pigs became hypoglycemic (blood glucose < 4 mmol/L), additional glucose was administered (20 ml à 30%). At timepoint 3 a baseline blood sample was collected and a bolus injection of ^15^N_2_-urea (2.3 mg/kg), 1-^13^C-glutamine (4.1 mg/kg), ring-^2^H_5_-phenylalanine (0.7 mg/kg) and 3,3-^2^H_2_-tyrosine (0.3 mg/kg) (Cambridge Isotopes Laboratories Inc. Tewksbury, MA, USA) was administered under 20 s. Thereafter, repeated blood samples were collected after 2, 4, 8, 16, 32, 40, 50, 60, 70, 80, 90, 100, 110 and 120 Minutes in EDTA tubes and saved on ice before centrifuging and then frozen at -80^◦^C until used.

### Clinical chemistry

Urine and plasma sodium (enzymatic sodium reagent, DZ114B-K01, Diazyme Laboratories, Poway, CA, USA), creatinine (enzymatic reagent, 8L24-31, Abbott Laboratories, Abbott Park, IL, USA), glucose (3L82-42, Abbott Laboratories) and urea (7D75-22, Abbott Laboratories) were analyzed on a BS430 chemistry analyzer (Mindray, Shenzhen, China). Plasma iohexol was measured with liquid chromatography (LiChrosorb RP18, 79915OD-574, Agilent, CA, USA). Iohexol clearance (IoCl) was calculated according to the formula:

$${\text{IoCl }}\left( {{\text{mGFR}}} \right) \, \left( {{\text{ml}}/{\text{min}}} \right) \, = {\text{ Infusion rate }}\left( {{\text{mg}}/{\text{min}}} \right)/{\text{ equilibrium concentration }}\left( {{\text{mg}}/{\text{ml}}} \right)$$

### Tracer analyses

Enrichments of the labelled urea (m + 2), glutamine (m + 1), phenylalanine (m + 5) and tyrosine (m + 2 and m + 4) were analyzed using gas chromatography–mass spectrometry as described before [23, 24]. Area under curve (AUC) of the enrichment decay was calculated and used to calculate the rate of appearance (Ra):

$$ {\text{Ra }} = {\text{ Bolus }}({\upmu}{\text{mol}}/{\text{kg}})/{\text{AUC}} $$

Whole body (WB) protein breakdown was estimated as the rate of appearance of phenylalanine (Ra(Phe)):

$${\text{WB protein breakdown }} = {\text{ Ra}}\left( {{\text{Phe}}} \right)$$

Whole body protein oxidation is calculated from the production of ^2^H_4_-tyrosine from ^2^H_5_phenylalnine:

$${\text{WB protein oxidation }} = {\text{ Ra}}\left( {{\text{Tyr}}} \right)*({\text{AUC}}\left( {^{{2}} {\text{H}}_{{4}} {\text{Tyr}}} \right)/{\text{AUC}}\left( {^{{2}} {\text{H}}_{{5}} {\text{Phe}}} \right)*({\text{Ra}}\left( {{\text{Phe}}} \right)/\left( {{\text{Ra}}\left( {{\text{Phe}}} \right) + {\text{Bolus}}\left( {{\text{Phe}}} \right)} \right)$$

Whole body protein synthesis is then calculated from the difference between protein breakdown and protein oxidation:

$${\text{WB protein synthesis }} = {\text{ WB protein breakdown }}{-}{\text{ WB protein oxidation}}.$$

### Analyses and physiologic parameters

Hemodynamic and respiratory parameters were documented at baseline and every hour for the following five hours duration of the experiment. SVV was monitored continuously, and values were documented hourly. Urine output was measured every hour, defined as 30 min before and 30 min after the timepoint of collecting urine samples for analyses (U-creatinine, U-urea, U-Na^+^, U-K^+^). At the same time points blood samples for plasma and arterial blood gas analyses were collected.

Creatinine clearance (CrCl) was calculated according to the formula:

$${\text{CrCl }} = \, \left( {{\text{U}} - {\text{creatinine }}*{\text{ U}} - {\text{volume}}} \right) \, / \, \left( {{\text{P}} - {\text{creatinine }}*{\text{ Time}}} \right)$$

Strong ion difference (SID) (abbreviated) was calculated as:

$${\text{SID }} = {\text{ S}} - {\text{Na}}^{ + } - {\text{ S}} - {\text{Cl}}^{ - }$$

### Statistical analysis

Data are presented as mean ± SD or median (IQR), pending distribution of data. Statistical significance was tested using Student’s T-test. The difference over time between the groups was tested using repeated measures ANOVA, allowing for non-linear evolution over time. Group differences for individual time points were tested using Student's T-test on á priori contrasts. Statistics were calculated using R (version 4.2.3). A p-value of < 0.05 was considered statistically significant.

## Results

At baseline, the two groups had comparable values on venous blood gas analyses drawn from the auricular vein (Table 1). The animals in the two groups did not differ in the amount of Na required to reach the hypernatremic state, the water group received Na 250 (167–250) mmol, and the no water group received Na 250 (167–292) mmol (p = 0.552). The water group received 1700 ± 300 ml of free water, during the experiment, and the overall fluid balance was 160 ± 370 ml in the water group and—810 ± 670 ml in the no water group (p = 0.005).

**Table 1:** Baseline characteristics, peripheral venous blood gas analyses

	Group	Mean ± SD	P value
pH	Water	7.43 ± 0.04	0.728
	No water	7.43 ± 0.04	
Hb (g/L)	Water	108 ± 6	0.903
	No water	109 ± 6	
K (mmol/L)	Water	4.0 ± 0.6	0.506
	No water	3.8 ± 0.1	
Na (mmol/L)	Water	139 ± 5	0.812
	No water	139 ± 4	
BE (mmol/L)	Water	4.0 ± 2.0	0.063
	No water	6.1 ± 1.8	
HCO_3_^−^ (mmol/L)	Water	27.7 ± 1.7	0.155
	No water	29.2 ± 1.9	

Baseline characteristics. Peripheral venous blood gas analyses. No difference between groups (water vs no water). Groups compared with t test

Both groups were stable in hemodynamic and respiratory parameters throughout the experiment. The no water group exhibited a significant increase in SVV during the experiment, indicating relative hypovolemia (Table 2).

**Table 2:** Respiratory and hemodynamic parameters

	Group	Baseline	T 1	T 2	T 3	T 4	T 5	P value
PaCO2 (kPa)	Water	5.2 ± 0.5	5.3 ± 0.5	5.2 ± 0.5	5.0 ± 0.4	5.1 ± 0.3	5.1 ± 0.3	0.062
	No water	5.2 ± 0.8	5.5 ± 0.9	5.3 ± 0.5	5.5 ± 0.6	5.5 ± 0.6	5.5 ± 0.7	
PaO_2_ / F_I_O_2_ (kPa)	Water	49.6 ± 11.2	53.5 ± 11.1	57.1 ± 10.3	57.7 ± 10.1	56.7 ± 8.8	55.8 ± 9.8	0.25
	No water	51.3 ± 12.2	54.5 ± 6.6	54.1 ± 7.0	52.7 ± 5.8	51.2 ± 6.2	48.0 ± 7.4	
PEEPtot (cmH_2_O)	Water	5.0 ± 0.0	5.0 ± 0.0	5.0 ± 0.0	5.0 ± 0.0	5.0 ± 0.0	5.0 ± 0.0	0.51
	No water	5.0 ± 0.4	4.9 ± 0.3	5.0 ± 0.1	5.1 ± 0.2	5.0 ± 0.0	5.3 ± 0.8	
Pplat (cmH_2_O)	Water	17 ± 3	15 ± 3	15 ± 3	15 ± 2	16 ± 3	16 ± 2	0.14
	No water	18 ± 3	16 ± 2	16 ± 2	17 ± 3	16 ± 2	18 ± 3	
MAP (mmHg)	Water	84 ± 15	75 ± 15	71 ± 9	71 ± 11	77 ± 17	77 ± 13	0.12
	No water	85 ± 8	69 ± 9	69 ± 10	75 ± 17	70 ± 12	69 ± 13	
HR (bpm)	Water	103 ± 16	95 ± 26	90 ± 19	87 ± 14	100 ± 24	98 ± 20	0.13
	No water	109 ± 34	100 ± 33	96 ± 23	103 ± 27	109 ± 29	113 ± 27	
SVV (%)	Water	7 ± 3	8 ± 2	7 ± 2	7 ± 1	8 ± 1	8 ± 2	2.7e-8
	No water	8 ± 2	13 ± 5	11 ± 5	12 ± 4	14 ± 4	16 ± 4	
CI (L/min/m^2^)	Water	5.5 ± 0.6	–	–	–	–	4.5 ± 0.6	0.72
	No water	5.2 ± 1.3	–	–	–	–	4.2 ± 0.5	
Temp (C°)	Water	39.8 ± 0.8	39.3 ± 0.7	39.1 ± 0.6	39.1 ± 0.5	39.2 ± 0.5	39.5 ± 0.7	0.004
	No water	38.8 ± 0.9	39.3 ± 0.7	39.7 ± 0.6	39.9 ± 0.6	40.1 ± 0.6	40.5 ± 0.9	

Pplat (Plateau pressure), MAP (Mean Arterial Pressure), HR (Heart Rate) bpm (beats per minute), SVV (Stroke Volume Variation), Cardiac Index (CI). Difference over time between the two groups (water vs no water) was tested using repeated measures ANOVA. Values expressed as mean ± SD

### Arterial blood gas analyses

**Fig. 1:**
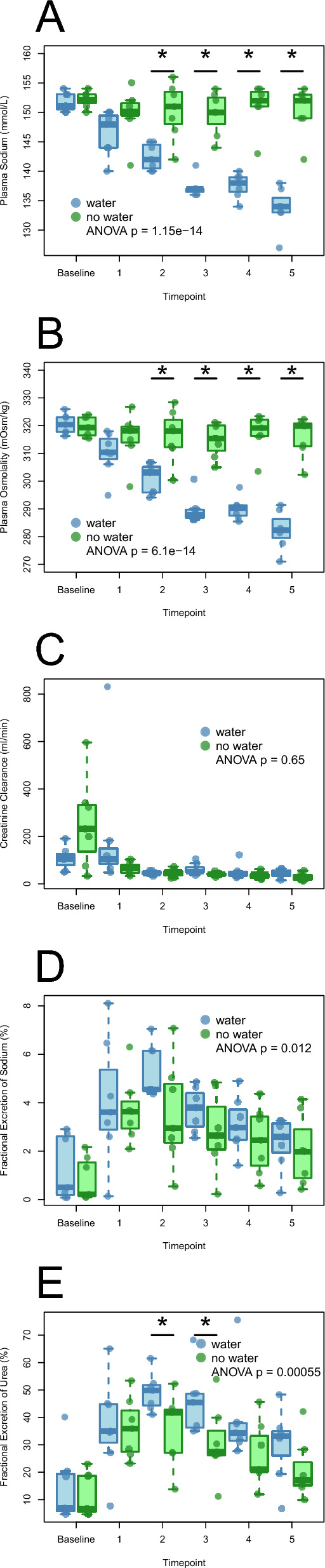
**A**–**E** Plasma sodium, plasma osmolality, Creatinine clearance, Fractional excretion of sodium and urea. Plasma concentrations of sodium (mmol/L) (**A**) and estimated osmolality in plasma (eOSM = 2Na + 2 K + glucose + urea in mOsm/kg) (**B**) from baseline and hourly throughout the protocol in the two groups. Creatinine clearance (ml/min) hourly in the two groups throughout the experiment (**C**). Fractional Excretion of Sodium (%) (**D**) and of urea (%) (**E**). Blue bars represent the water group and green bars represent the no water group. A p value < 0.05 is considered statistically significant and marked*

Plasma sodium levels normalized after two hours in the water group (142 ± 2 mmol/l), whereas the no water group was hypernatremic with elevated plasma levels throughout the experiment (Fig. 1A). Plasma potassium was within the normal range in both groups during the experiment but increased slightly in the no water group. pH increased in the water group, which developed a discrete metabolic alkalosis throughout the experiment. SID changed similarly in the two groups during the experiment. Levels of Hemoglobin (Hb) increased in the no water group as compared to the water group, as well as lactate. Blood glucose decreased significantly in the no water group with similar amounts of glucose administered in the two groups (water group 12 ± 5 ml vs 17 ± 12 in the no water group, p = 0.438), whereas Base Excess and HCO_3_^−^ increased in the water group (Table 3).

**Table 3:** Arterial blood gas analyses

	Group	Baseline	T 1	T 2	T 3	T 4	T 5	P value
K (mmol/L)	Water	4.3 ± 0.2	3.9 ± 0.4	3.8 ± 0.3	3.8 ± 0.3	4.0 ± 0.2	3.9 ± 0.2	0.00027
	No water	4.0 ± 0.3	4.1 ± 0.3	4.2 ± 0.3	4.2 ± 0.3	4.4 ± 0.3	4.4 ± 0.3	
pH	Water	7.46 ± 0.03	7.47 ± 0.04	7.48 ± 0.04	7.49 ± 0.04	7.48 ± 0.05	7.48 ± 0.04	0.0028
	No water	7.46 ± 0.05	7.44 ± 0.07	7.44 ± 0.05	7.44 ± 0.04	7.44 ± 0.04	7.43 ± 0.05	
Hb (g/L)	Water	91 ± 5	104 ± 8	103 ± 10	101 ± 11	104 ± 16	101 ± 14	0.011
	No water	94 ± 7	107 ± 10	107 ± 8	112 ± 10	112 ± 10	111 ± 10	
Blood Glucose (mmol/L)	Water	6.3 ± 1.2	6.9 ± 2.8	6.0 ± 1.9	5.0 ± 1.8	4.4 ± 2.0	4.3 ± 2.0	0.011
	No water	5.2 ± 1.5	5.7 ± 1.7	4.9 ± 1.8	3.9 ± 1.9	3.4 ± 1.7	3.1 ± 1.9	
Lactate (mmol/L)	Water	1.0 ± 0.4	1.7 ± 0.7	1.7 ± 1.0	1.7 ± 1.4	2.0 ± 1.7	2.2 ± 1.2	0.0046
	No water	0.9 ± 0.2	2.3 ± 0.8	2.5 ± 0.8	2.6 ± 0.7	2.7 ± 0.9	2.9 ± 0.9	
BE (mmol/L)	Water	3.3 ± 0.9	4.2 ± 1.4	4.8 ± 1.6	5.1 ± 2.2	4.7 ± 2.8	5.0 ± 2.8	0.012
	No water	3.3 ± 2.0	3.5 ± 1.9	3.1 ± 2.2	3.6 ± 2.2	3.3 ± 2.5	2.6 ± 3.1	
HCO_3_ (mmol/L)	Water	27.4 ± 0.9	28.3 ± 1.4	28.8 ± 1.5	29.1 ± 2.0	28.8 ± 2.6	29.1 ± 2.6	0.007
	No water	27.5 ± 1.8	27.4 ± 1.8	27.2 ± 2.1	27.6 ± 2.0	27.4 ± 2.3	26.6 ± 2.8	
SID	Water	38 ± 3	35 ± 5	38 ± 4	36 ± 3	41 ± 2	38 ± 3	0.43
	No water	37 ± 4	36 ± 4	38 ± 7	38 ± 4	42 ± 3	40 ± 4	
Na (mmol/L)	Water No water	152 ± 2 152 ± 1	146 ± 4 150 ± 4	142 ± 2 150 ± 5	137 ± 2 150 ± 4	138 ± 2 151 ± 4	134 ± 4 150 ± 4	< 0.001
Cl (mmol/L)	Water No water	114 ± 3 115 ± 4	111 ± 3 114 ± 2	105 ± 3 113 ± 4	101 ± 3 111 ± 3	96 ± 2 109 ± 3	95 ± 2 110 ± 3	< 0.001

Hemoglobin (Hb), Strong Ion Difference (SID). Difference over time between the two groups (water vs no water) was tested using repeated measures ANOVA. Values expressed as mean ± SD

### Analyses of osmolality and measurement of kidney function

**Fig. 2:**
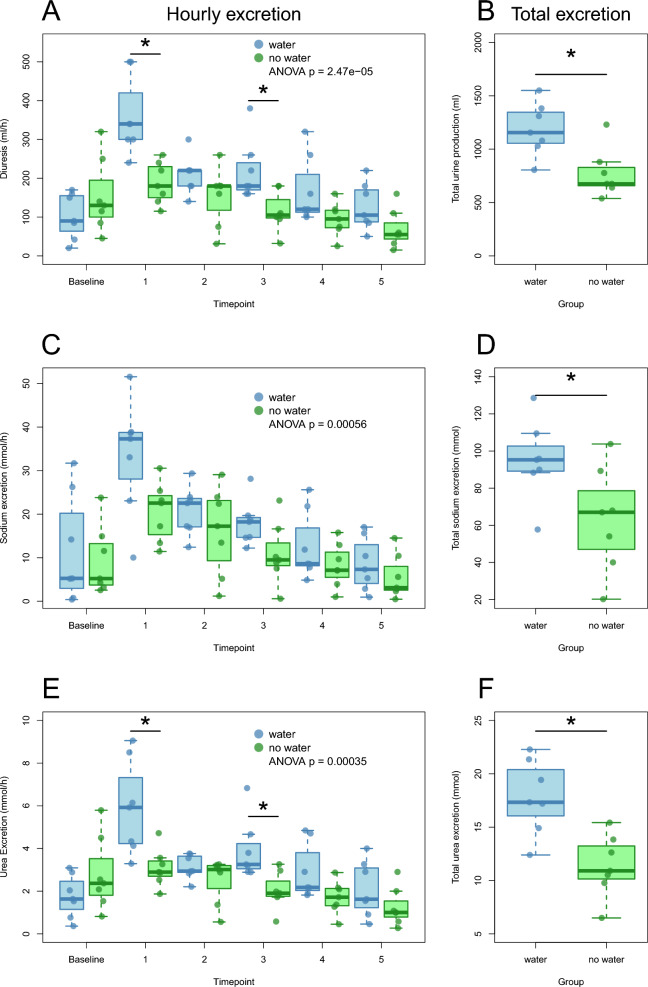
**A**–**F** Diuresis and excretion of sodium and urea. Hourly diuresis (ml/h) from baseline and throughout the experiment in the two groups (**A**), total urine production in the two groups (**B**). Hourly sodium excretion (mmol/) in the two groups throughout the experiment (**C**) and total sodium excretion (**D**). Hourly urea excretion (mmol/h) throughout the experiment in the two groups (**E**) and total urea excretion (**F**). Blue bars represent the water group and green bars represent the no water group. A p value < 0.05 is considered statistically significant and marked*

Estimated osmolality (eOSM) was calculated as eOSM = 2Na + 2 K + glucose + urea. eOSM decreased in the water group during the experiment, whereas the no water group maintained the hyperosmolality induced at baseline (Fig. 1B). Diuresis was higher in the water group than in the no water group (Fig. 2A). Plasma creatinine and plasma urea were comparable in the two groups at baseline, both markers increased in the no water group but not in the water group during the experiment (Table 4).

**Table 4:** Analyses of kidney function

	Group	Baseline	T 1	T 2	T 3	T 4	T 5	P value
Plasma Crea (µmol/L)	Water	69 ± 4	72 ± 3	73 ± 5	73 ± 6	76 ± 17	72 ± 8	0.026
	No water	66 ± 15	72 ± 16	76 ± 17	82 ± 19	88 ± 19	97 ± 18	
Plasma Urea (mmol/L)	Water	2.3 ± 0.6	2.3 ± 0.5	2.4 ± 0.5	2.4 ± 0.4	2.7 ± 0.7	2.7 ± 0.4	0.012
	No water	2.2 ± 0.4	2.4 ± 0.4	2.5 ± 0.4	2.7 ± 0.4	3.0 ± 0.4	3.4 ± 0.4	
Urine Na (mmol/L)	Water No water	86 ± 67 72 ± 59	94 ± 32 109 ± 15	99 ± 7 94 ± 33	84 ± 12 84 ± 33	73 ± 15 80 ± 27	63 ± 27 71 ± 29	0.810

Difference over time between the two groups (water vs no water) was tested using repeated measures ANOVA. Values expressed as mean ± SD

Fractional excretion of sodium and urea was higher in the water group as compared to the no water group, as well as total excretion of sodium (Fig. 1D, E, Fig. 2C–F). Sodium excretion during the experiment after induction of hypernatremia was higher in the water group as compared to the no water group (Figs. 2C, D), as was urea excretion (Fig. 2E, F). Creatinine clearance was similar in the two groups during the experiment (Fig. 1C). Iohexol clearance (mGFR) reached a steady state in the water group at timepoint 3 with mean values of 46 ± 4 ml/min, whereas the no water group had a continuous decrease in mGFR throughout the experiment, from 56 ± 10 ml/min to 40 ± 8 ml/min, corresponding to a decrease of -26% ± 12% in mGFR.

### Metabolite production rates

Production rates (Ra) of glutamine (Gln) were higher in the no water group than in the water group, whereas production rates of urea were not significantly different between the groups. Whole body protein breakdown, synthesis and oxidation rates were comparable in the two groups (Table 5).

**Table 5:** Stable isotope tracers and metabolite production rates

	Group		P value
Ra Urea (μmol/kg/h)	Water	1387 ± 418	0.524
	No water	1563 ± 572	
Ra glutamine (μmol/kg/h)	Water	1084 ± 110	0.029*
	No water	1305 ± 209	
Protein Breakdown (μmol Phe/kg/h)	Water	189 ± 17	0.202
	No water	202 ± 18	
Protein oxidaton (μmol Phe/kg/h)	Water	15 ± 2	0.072
	No water	17 ± 3	
Protein synthesis (μmol Phe/kg/h)	Water	174 ± 19	0.306
	No water	184 ± 18	

p values from individual independent t-test, Bonferroni correction not applied. Values expressed as mean ± SD

## Discussion

The main finding of the present study was that free water infusion, aiming for normonatremia, improved sodium excretion in pigs with sodium chloride induced hypernatremia. The increase in sodium excretion could be explained by a decrease in tubular reabsorption, as reflected by an increase in fractional excretion of sodium and to some extent, a higher GFR, even though the wide spread is likely to influence the results.

We also investigated the effect of hypernatremia on amino acid and protein metabolism. In hypertonicity, organic osmolytes are upregulated intracellularly to maintain cell volume [3, 4]. This shift to production of organic osmolytes in response to dehydration is consistent with the aestivation response. The aestivation response is a water-conserving mechanism which relies on urea production by the liver and urea recycling by the kidneys. The urea production in the liver includes urea synthesis from amino acids, with consequent muscle breakdown. Urea is an important mechanism to keep up the reabsorption in the kidney [25–27]. The aestivation response is enhanced in more severe illness [5, 28].

In the present study, we measured the appearance rates (Ra) of different metabolites in plasma. The Ra of urea (equal to de novo production of urea) was comparable in the two groups, demonstrating that the increased plasma levels of urea in the no water group were not the result of an increased production of urea, but due to a decrease in urinary clearance. The comparable Ra of urea and protein breakdown in the two groups might indicate that the protocol of the present study was too short for the aestivation response to manifest, since no increase in protein breakdown and urea synthesis was demonstrated. The increased Ra of glutamine in plasma in the no water group is consistent with elevated de novo production and protein breakdown in this group. Intracellular glutamine metabolism is influenced by hyperosmolality induced volume changes [29, 30]. It is the most common free amino acid, predominantly produced in skeletal muscle [31]. Thus, the amino acids in the cytoplasm contribute to the cellular water balance, while glutamine in plasma serve as a nitrogen shuttle from muscle to liver, where urea is produced. The present study supports glutamine production as a feature to maintain cell volume during hypertonicity.

The animals were stable in respiratory and hemodynamic parameters throughout the experiment. The no water group had an overall negative fluid balance, in comparison with the water group that had a slightly positive fluid balance, further the no water group showed signs of hypovolemia, as indicated by increased SVV. These findings might have contributed to aldosterone-induced sodium retention, and thereby a reduced sodium output in this group. For future experiments the titration of free water, in order to find the optimal window for fluid and sodium balance and the mobilization of both should be considered. This would imply a longer experiment with the administration of larger volumes of isotonic saline in order to better reflect the clinical scenario after resuscitation in critical illness. The water group developed a discrete metabolic alkalosis during the experiment; while this was not explained by SID, a tendency of lower PaCO_2_ in the water group might have contributed.

The design of the present study could discern the effect of administration of free water in addition to furosemide infusion and the overall effect on sodium mobilization and normalization of hypernatremic hyperosmolality. This is important since patients with higher eOSM are weaker and since aggressive de-resuscitation without a concomitant decrease in eOSM via water administration contributes to a prolonged hyperosmolar state, higher concentrations of organic osmolytes, protein breakdown, muscle consumption and results in muscle weakness [12].

Even though the concentrating capacity of the porcine kidney is similar to humans [32] and the natriuretic effect of furosemide in pigs is well studied [33, 34], the possibility of interspecies variability cannot be excluded. Another limitation of the study is the relatively short duration. Although the furosemide effect is prompt and five hours is enough to investigate the study question [35], the sodium loading and de-resuscitation phases in critical illness is often extended over days and weeks. It is likely that the rapidly induced hypernatremia has a somewhat different response than the prolonged infusions of isotonic crystalloids followed by diuretic treatment and slow development of hyperosmolality normally seen in the clinical setting. However, the electrolyte disturbance is meant to model a late resuscitation stage where sodium, rather than water mobilization is warranted. In critically ill patients, renal creatinine clearance is not always a good estimate of measured GFR using iohexol [36]. In the present study creatinine clearance was comparable in the two groups throughout the experiment, while mGFR calculated on iohexol clearance showed that the water group reached a steady state at three hours [37, 38], a finding that indicates maintained filtration in the water group, whereas the no water group had continuously decreasing mGFR throughout the experiment indicating that no steady state was achieved.

The use of diuretics [39] has not been shown to improve outcome, this might be attributed to the hypernatremia that it causes. Our results indicate that maintaining normo-natremia is feasible and that addition of free water concomitantly with diuretics promotes sodium mobilization. The net effect of simultaneous addition of free water and water removal whilst maintaining normo-natremia needs to be explored in further studies in order to determine if this is translated to improved patient outcome.

In conclusion, in the present study, we demonstrated that the addition of free water to furosemide for sodium and water mobilization in hypernatremia enhances sodium excretion. The results also indicate that glutamine production intracellularly could be a key feature to maintain cell volume during hypertonicity.

## Data Availability

The data that support the findings of this study are available from the corresponding author (ABT) upon reasonable request.
